# Biochemical and Microstructural Characteristics of Collagen Biopolymer from Unicornfish (*Naso reticulatus* Randall, 2001) Bone Prepared with Various Acid Types

**DOI:** 10.3390/polym15041054

**Published:** 2023-02-20

**Authors:** Nurul Syazwanie Fatiroi, Abdul Aziz Jaziri, Rossita Shapawi, Ruzaidi Azli Mohd Mokhtar, Wan Norhana Md. Noordin, Nurul Huda

**Affiliations:** 1 Faculty of Food Science and Nutrition, Universiti Malaysia Sabah, Kota Kinabalu 88400, Sabah, Malaysia; 2 Faculty of Fisheries and Marine Science, Universitas Brawijaya, Malang 65145, East Java, Indonesia; 3 Borneo Marine Research Institute, Universiti Malaysia Sabah, Kota Kinabalu 88400, Sabah, Malaysia; 4 Biotechnology Research Institute, Universiti Malaysia Sabah, Kota Kinabalu 88400, Sabah, Malaysia; 5 Fisheries Research Institute, Batu Maung 11960, Penang, Malaysia; 6 Faculty of Sustainable Agriculture, Universiti Malaysia Sabah, Sandakan 90509, Sabah, Malaysia

**Keywords:** collagen biopolymer, unicornfish bone, acid extraction, characterization

## Abstract

Biopolymer-like collagen has great industrial potential in terms of its excellent properties, such as strong biocompatibility, high degradability, and low antigenicity. Collagen derived from fish by-products is preferable as it is safer (free from transmittable diseases) and acceptable to most religious beliefs. This study aimed to characterize the unicornfish (*Naso reticulatus* Randall, 2001) bone collagens prepared with different type of acids, i.e., acetic acid, lactic acid, and citric acid. A higher yield (Y) (*p* < 0.05) was obtained in the citric-acid-soluble collagen (CASC) (Y = 1.36%), followed by the lactic-acid-soluble collagen (LASC) (Y = 1.08%) and acetic-acid-soluble collagen (AASC) (Y = 0.40%). All extracted collagens were classified as type I due to the presence of 2-alpha chains (α1 and α2). Their prominent absorption spectra were located at the wavelengths of 229.83 nm to 231.17 nm. This is similar to wavelengths reported for other fish collagens. The X-ray diffraction (XRD) and infrared (IR) data demonstrated that the triple-helical structure of type I collagens was still preserved after the acid-extraction process. In terms of thermal stability, all samples had similar maximum transition temperatures (*T_max_* = 33.34–33.51 °C). A higher relative solubility (RS) of the unicornfish bone collagens was observed at low salt concentration (0–10 g/L) (RS > 80%) and at acidic condition (pH 1.0 to pH 3.0) (RS > 75%). The extracted collagen samples had an irregular and dense flake structure with random coiled filaments. Overall, bones of unicornfish may be used as a substitute source of collagen.

## 1. Introduction

Biopolymer collagen, a fibrillar protein, is one of the main structural components in the connective tissues of mammals and makes up almost 30% of total protein composition [1]. It is characterized by the unique right-handed triple-helical structure, composed of three left-handed polyproline-like helices, each with a (Gly–Xa–Ya) repeating sequence where Xa and Ya are often proline and hydroxyproline [2]. At least 29 types (I–XXIX) of collagens with different structures of polypeptides, amino acid motifs, and molecular characteristics have been studied [3]. Type I collagen has been intensively explored by researchers because of its special traits (i.e., strong biocompatibility, high biodegradability, and lack of antigenicity) [4]. Collagen has great potential in various industrial sectors such as medical, pharmaceutical, nutraceutical, and cosmetic. It has been approved for use in tissue engineering due to its ability to stimulate cellular migration, tissue matrix interaction, and tissue regeneration [5,6]. It is also applicable in developing drug-delivery systems and in treating hypertension, obesity, and diabetes [7]. In addition, it is an important cosmetic ingredient that serves as a natural humectant and moisturizer, preventing aging of the skin [8]. In food manufacturing, collagen is often used as a colloidal stabilizer, emulsifier, and foaming agent [9]. Collagens are mostly derived from the skins and bones of land vertebrates, especially bovine and porcine vertebrates. However, use of these animals raises consumer apprehension due to reported infectious diseases such as bovine spongiform encephalopathy, transmissible spongiform encephalopathy, and foot-and-mouth disease. Another constraint is associated with religious belief. For instance, Muslims and Jews are prohibited to consume or use porcine-derivative products, while cows and beef derivatives are forbidden in Hinduism [10]. To deal with these issues, alternative sources of collagen are necessary.

Over the last decade, fish collagen has gained considerable attention amongst scientists. This is evidenced by the large number of publications related to the extraction of fish collagens. Sources of the collagens include tiger grouper (*Epinephelus fuscoguttatus*) skin [11], bigeye tuna (*Thunnus obesus*) skin, bone, and scale [12], lizardfish (*Saurida tumbil*) skin, bone, and scale [13,14,15], grass carp (*Ctenopharyngodon idellus*) skin, bone, and scale [16], golden pompano (*Trachinotus blochii*) skin and bone [17], Spanish mackerel (*Scomberomorous niphonius*) skin and bone [18], sturgeon (*Huso huso*) skin [19], puffer fish (*Lagocephalus inermis*) skin [20], red stingray (*Dasytis akajei*) skin [21], Siberian sturgeon (*Acipenser baerii*) cartilage [22], leather jacket (*Odonus niger*) bone [23], tilapia (*Oreochromis mossambicus*) bone [24], grey mullet (*Mugil cephalus*) scale [25], and yellow tuna (*thunnus albacore*) swim bladders [26]. Fish collagens are mostly categorized as type I and their physicochemical properties, including thermal stability, solubility, and triple-helical structures, have also been evaluated. Interestingly, few studies have demonstrated that modification of the extraction process could increase the thermostability of fish collagen [27].

Unicornfish (*Naso reticulatus* Randall, 2001) belongs to the family Acanthuridae. It has a convex head with slight angularity before its eye, no horn on the forehead, and an emarginated caudal fin. This fish mainly lives in tandem with coral reefs, feeds on algae, and has tight skin-like jacket [28]. It is popularly served grilled in restaurants. By-products from *N. reticulatus* are usually discarded after processing, resulting in loss of a valuable biological resource. Therefore, converting the by-products into high-end product such as collagen is highly beneficial. Moreover, there is little information regarding extraction of collagen from this fish. Hence, this work aimed to compare the extractability of collagen from unicornfish bone using different acids (i.e., acetic acid, lactic acid, and citric acid), and determine the physicochemical and structural properties of the collagens.

## 2. Results and Discussion

### 2.1. Yield and Hydroxyproline Content of Acid-Soluble Collagens

Table 1 shows the yields (%) of acid-soluble collagens derived from unicornfish bone. The highest yield (Y) was recorded in CASC (Y = 1.36%) (*p* < 0.05) compared to AASC (Y = 0.40%) and LASC (Y = 1.08%) suggesting that citric acid may be the most effective acid for extracting collagen from fish bones. This finding was in agreement with the previous report on collagen from lizardfish (*S. tumbil*) bone [13]. The results in the present study were also comparable to those for other acid-soluble fish bone collagens such as bigeye tuna (*T. obesus*) (Y = 0.1%) [12], tilapia (*O. niloticus*) (Y = 0.5%) [24], grass carp (*C. idellus*) (Y = 0.7%) [16], carp (*C. carpio*) (Y = 1.06%) [25], and golden pompano (*T. blochii*) (Y = 1.25%) [17]. Logically different fish species, acids, and extraction procedures might influence the collagen yields [14,15]. There was no significant difference in hydroxyproline (Hyp) content (*p* > 0.05) between AASC and LASC. However, the Hyp content in CASC was significantly lower (*p* < 0.05). The Hyp contents recorded in the present study were lower than those for bigeye tuna (*T. obesus*) (82–87 mg/g) [12], cobia (*Rachycentron canadum*) (84–99 mg/g) [29], and marine eel (*Evencheslys macrura*) (94–98 mg/g) [30] bone collagens. These differences could be attributed to several factors including type of species, size, age, structure, and composition of fish tissue, as well as the extraction process [31]. Collagen with high Hyp content could help to improve the structural stability of its molecules. Kittiphattanabawon et al. [32] stated that Hyp is a prominent component of amino acids and plays an essential role in stabilizing the triple-helical structure of collagen.

### 2.2. Color Attributes

Color attributes of the AASC, LASC, and CASC are presented in Table 1. Sadowska et al. [33] pointed out that a lighter-color collagen is more preferable for developing new products in food, nutraceutical, or medical applications because it does not alter the original color of a product. There were no significant differences (*p* > 0.05) in the lightness (L*) and whiteness (WI) values of all extracted collagens in the present study. However, significantly higher L* values ranging from 79.25 to 82.55 was noted in the unicornfish bone collagens compared to lizardfish (*S. tumbil*) (72.76) [15] and barramundi (*Lates calcalifer*) (44.76–65.41) skin collagens [34]. Addition of hydrogen peroxide (H_2_O_2_) could increase the lightness of acid extracted collagens, as reported in lizardfish (*S. tumbil*) bone (88.54) [13] and snakehead fish (*Channa argus*) skin (89.49) [35] collagens so there is potential for further experimentation with different H_2_O_2_ concentrations. Moreover, the a* and b* values of LASC were significantly higher (*p* < 0.05) than those of AASC and CASC.

### 2.3. SDS-PAGE Profile

**Figure 1:**
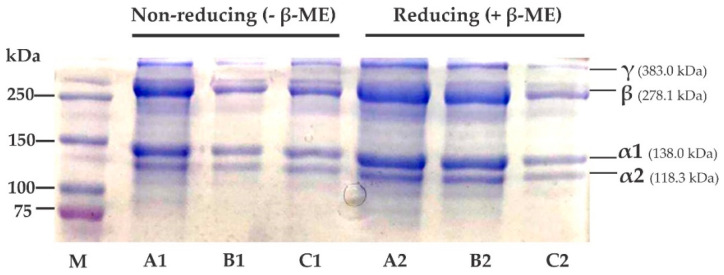
SDS-PAGE image of acid soluble unicornfish bone collagen. M: protein marker; A1 and A2: acetic acid soluble collagen (AASC); B1 and B2: lactic acid soluble collagen (LASC); C1 and C2: citric acid soluble collagen (CASC).

An SDS-PAGE image of the unicornfish bone collagens is presented in Figure 1. The electrophoretic pattern of each sample was almost similar, with two alpha (α1 and α2), one beta (β), and one gamma (γ) chains. The molecular weight (MW) of each alpha chain was estimated as 138.0 kDa and 118.3 kDa, respectively. Benjakul et al. [36] suggested that type I collagen was characterized by the presence of two alpha chains (α1 and α2). Based on this, unicornfish bone collagens were also categorized as type I. All acid-soluble collagens assessed in this study were comparable to previous literature on type I collagen fish collagen from seabass (*L. calcarifer*) (α1 = 118 kDa and α2 = 105 kDa) [37], loach (*M. anguillicaudatus*) (α1 = 127 kDa and α2 = 115 kDa) [38], golden pompano (*T. blochii*) (α1 = 120 kDa and α2 = 100 kDa) [17], and Nile tilapia (O. *niloticus*) (α1 = 125 kDa and α2 = 114 kDa) [39]. Other electrophoretic chains found in all extracted collagens (i.e., β = 278.1 kDa and γ = 383.0 kDa), indicate dimer and trimer bands as observed in our previous findings on lizardfish (*S. tumbil*) fish collagens [13,14,15]. Further analysis with addition of β-ME (reducing) and without β-ME (non-reducing), showed no differences in electrophoretic patterns of AASC, LASC, or CASC, and absence of disulfide bonds, as mentioned in previous literature [13,14,15].

### 2.4. UV Absorption Spectra

**Figure 2:**
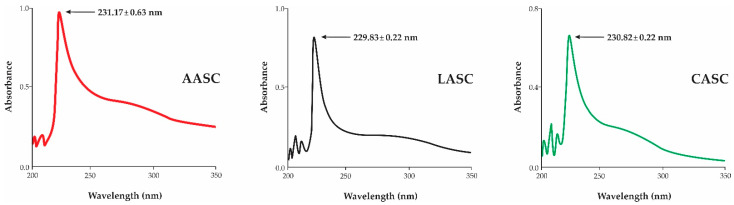
UV absorption spectra of acid soluble unicornfish (*N. reticulatus*) bone collagen. AASC: acetic acid soluble collagen; LASC: lactic acid- soluble collagen; CASC: citric acid soluble collagen.

Figure 2 presents the UV absorption spectra of the AASC, LASC, and CASC. In general, a prominent spectrum of fish collagen was located at the wavelengths of 210 nm to 240 nm [40]. All acid-soluble collagens from the bone of unicornfish were within the maximum spectral ranges proposed by previous works, with no significant differences (*p* > 0.05) among the samples. The highest peak observed in this study was in accordance with other fish collagens, such as Siberian sturgeon (*A. baerii*) [22], red drum (*Sciaenops ocellatus*) [41], lizardfish (*S. tumbil*) [13], and puffer fish (*L. inermis*) [20]. The spectra observed were associated with the functional groups of carboxyl (-COOH), carbonyl (C=O), and amides (CONH_2_), which belong to the polypeptide chains of fish collagen, as proposed by Jaziri et al. [13]. Other low absorption peaks (300 nm to 250 nm) were also observed in all extracted collagens and were likely related to the aromatic amino acids, such as phenylalanine, tryptophan, and tyrosine. It is therefore assumed that collagens extracted from the bone of unicornfish contain low composition of aromatic amino acids.

### 2.5. Attenuated Total Reflection–Fourier Transform Infrared Spectroscopy (ATR–FTIR)

**Figure 3:**
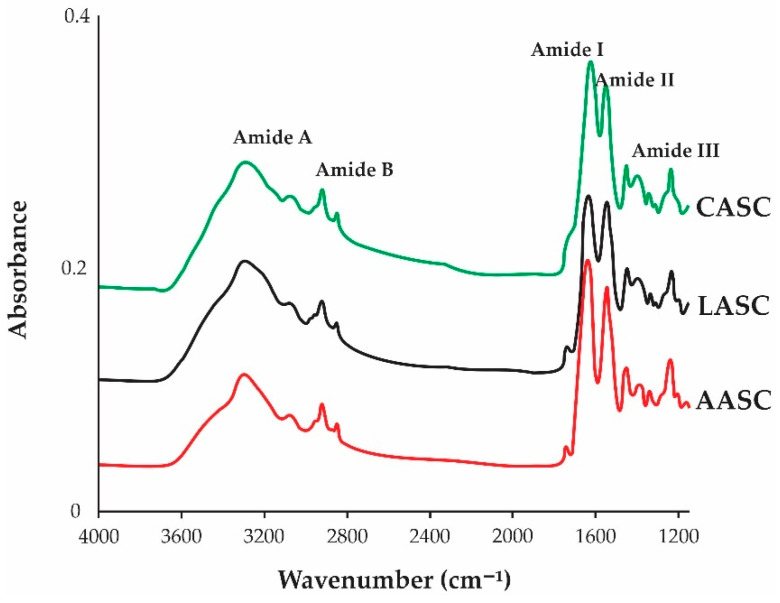
IR spectra of acid soluble unicornfish (*N. reticulatus*) bone collagen. AASC: acetic acid soluble collagen; LASC: lactic acid soluble collagen; CASC: citric acid soluble collagen.

FTIR spectra of AASC, LASC, and CASC are shown in Figure 3. Five significant peaks (Amides: A, B, I, II, and III) were clearly identified in all samples. As described in Table 2, Amide A represents the N-H stretching vibrations with hydrogen bonds which represents the protein molecules and is usually located at 3200–3440 cm^–1^ region [42], as observed in the present study. For Amide B, it described an asymmetric stretching of CH_2_ vibrations [21], and the higher wavenumber region of Amide B was observed in the LASC and CASC samples. Meanwhile, Amide I is often related to the secondary structure of proteins, with wavenumbers ranging from 1600 to 1700 cm^–1^ [43] and represents the stretching vibration of the backbone carbonyl group (C=O) in polypeptides. The strong bands of Amide I in all acid-soluble collagens in the present study were in accordance with other literature [23]. On the other hand, Amides II and III have been widely used for the identification of triple-helical structure of collagen [44]. Amide II, typically located at the wavenumbers from 1500 cm^–1^ to 1600 cm^–1^ [45], which correspond to the N–H bending vibrations combined with the C–N stretching vibrations. The CASC showed lower wavenumbers compared to the AASC and LASC, indicating more H bonds in the CASC. Meanwhile, Amide III reflected the peak combination between C–N stretching and N–H remodeling, resulting in amide linkages which generally occur between 1200 cm^–1^ and 1350 cm^–1^ [41]. Similar peaks were also noted in other fish collagens, such as Nile tilapia (*O. niloticus*) [39], lizardfish (*S. tumbil*) [13,14,15], purple spotted bigeye snapper (*P. tayenus*) [46], sturgeon fish (*H. huso*) [19], barramundi (*L. calcarifer*) [37], and loach (*M. anguilllicaudatus*) [38].

In terms of stability of the triple-helical structures, Benjakul et al. [36] suggested that the triple-helical structure was preserved if the difference in wavenumber between Amides I and II (Δ*v = v_I−_v_II_*) was less than 100 cm^−1^ [47]. Based on this guideline, our results confirmed that the triple-helical structure of all extracted collagens from unicornfish bone were maintained because Δ*v* of the AASC, LASC, and CASC were 95.05 cm^–1^, 93.19 cm^–1^, and 76.42 cm^–1^, respectively. Another approach is through the ratio of the Amide III and 1450 cm^−1^ band (AIII/A1450), as proposed by Doyle et al. [48]. After validation, the triple-helical structures of extracted collagens did not change during the extraction process as described from their absorption ratio values (~1.0), suggesting that the use of acetic, citric, and lactic acids during the extraction process could solubilize collagens without damaging the structures.

### 2.6. Evaluation of X-ray Diffraction (XRD)

Table 3 presents the diffraction data of the AASC, LASC, and CASC. Generally, two significant peaks were observed. The first peak was sharp, and second peak was broader. The obtained diffraction data were comparable to the triple-helical structure of calf-skin collagen (standard) [15]. Similar diffraction motifs were also noted in tilapia (*O. niloticus*) skin [49], carp fish (*C. carpio*) scale [24], golden pompano (*T. blochii*) skin and bone [27] and lizardfish (*S. tumbil*) skin, scale, and bone [13,14,15] collagens. In order to predict the minimum value of repeated spacings d (Å), the Bragg formula by Zhang et al. [25]. was used with d(Å) = λ/2sin *θ* (where λ is the X-ray wavelength of 1.54 Å and *θ* is the Bragg diffraction angle). As shown in Table 3, the first peak (d = 1.13–1.14 nm) reflects the distance between the molecular chains of triple-helical structure found in fish collagen, with higher d values being detected in the AASC and LASC samples. Meanwhile, the d value of AASC, LASC, and CASC samples in the second highest peak ranged from 0.33 nm to 0.34 nm, with the lowest observed in the CASC. This peak denotes the distance between skeletons of fish-collagen structure. The diameter (d) of a collagen molecule with a single left-handed helix chain and a triple-helical structure was consistent with the diameter of collagen from the barracuda skin prepared by solubilizing with different acids [13]. Overall, our extracted collagens showed no denaturation in the triple-helical structures and were in their native conformations.

### 2.7. Thermostability of Acid-Soluble Collagen

Thermostability, as determined by *T_max_* value of the AASC, LASC, and CASC, is listed in Table 3. A higher thermostability was demonstrated in the AASC (*T_max_* = 33.51 °C), followed by LASC (*T_max_* = 33.39 °C) and CASC (*T_max_* = 33.34 °C. According to Benjakul et al. [36], thermostability of collagen was related to the presence of amino acids (proline and hydroxyproline), particularly at pyrrolidine rings that are governed by the H bonding via the hydroxyl group of Hyp. In addition, Hyp served as stabilizer of the triple-helical structure through H bonding in coil-coiled alpha chains [50]. Our *T_max_* results (around 33 °C) were comparable to other fish bone collagens, such as purple-spotted bigeye (*P. tayenus*) (30.80–31.48 °C) [32], Siberian sturgeon (*A. baerii*) skin (28.30 °C) [22], grass carp (*C. idellus*) (36 °C) [16], and golden pompano (*T. blochii*) skin (38.23 °C) [17]. Interestingly, fish from tropical waters showed higher thermostability compared to temperate fish such as Spanish mackerel (*S. niphonius*) (18.02 °C) [18]. The delta H value (Δ*H*) was defined as the area located under the thermogram peaks, which reflects the energy required to uncouple the alpha chains of collagen and convert them into random coils. The Δ*H* of the AASC sample was lower than of the LASC and CASC samples, indicating a lower energy used in AASC. The differences in the *T_max_* and Δ*H* values of fish collagen were likely influenced by many factors, such as the composition of amino acids, extraction process, fish species, and other factors, particularly water temperature and habitat [32].

### 2.8. Microstructure Profile

**Figure 4:**
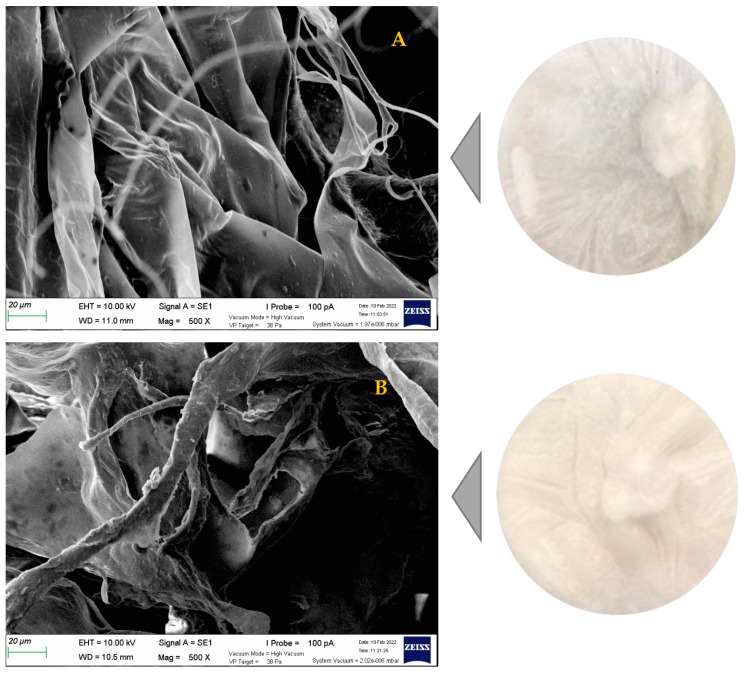

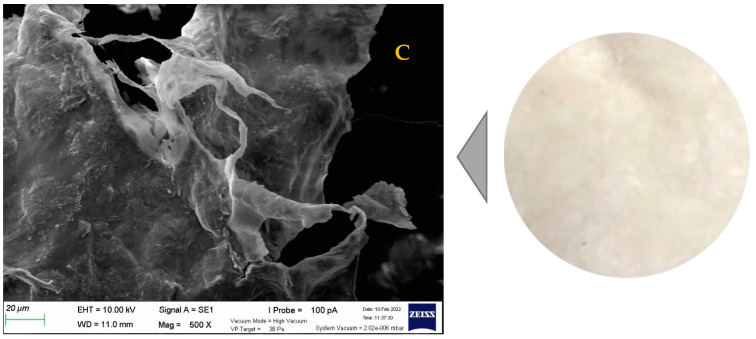
SEM image (magnification 500×) of the acid-soluble unicornfish (*N. reticulatus*) bone collagen. (**A**) acetic acid soluble collagen (AASC); (**B**) lactic acid soluble collagen (LASC); and (**C**) citric acid soluble collagen (CASC).

The AASC, LASC, and CASC were scanned under a scanning electron microscope (SEM), and the morphological structures were appraised. As illustrated in Figure 4, the extracted collagens of unicornfish bones showed fibril-forming structures, multi-layered forms, and irregular sheet-like films linked by random-coiled filaments. In addition, the wrinkled and porous structures were also clearly visible at magnification of 500×, indicating the samples were dehydrated during the lyophilization process, as documented by Schuetz et al. [51]. According to Lim et al. [7], fish collagen with fibrillary, interconnectivity, and sheet-like film structures could be a potential source of biomaterials for nutraceutical, pharmaceutical, and biomedical products to be used in wound dressing, skin and bone tissue formation, cell migration, and coating material. The microstructure profiles of Lizardfish (*S. tumbil*) skin, bone, and scale [13,14,15], miiuy croaker (*M. miiuy*) scale [52], black ruff (*Centrolophus niger*) skin [53], and marine eel (*E. macrura*) skin [30] collagens were in agreement with this recent work.

### 2.9. Solubility Studies

**Figure 5:**
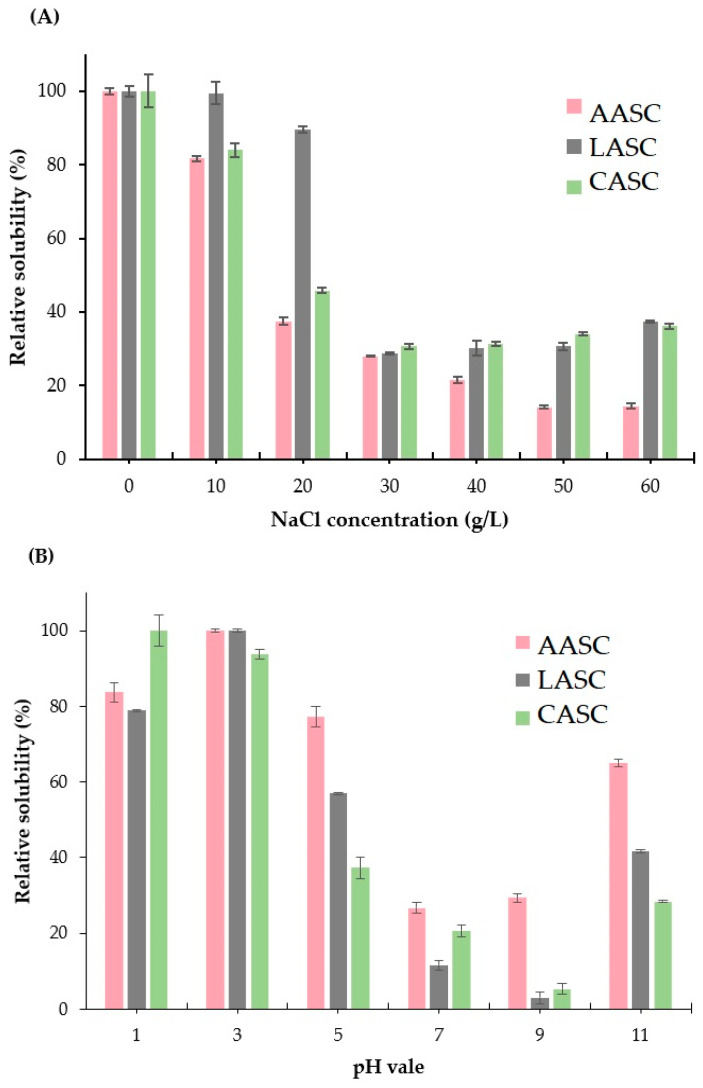
Relative solubility (RS) of acid soluble collagens from the unicornfish (*N. reticulatus*) bone at (**A**) different NaCl concentrations and (**B**) various pH levels. AASC: acetic acid soluble collagen; LASC: lactic acid soluble collagen; CASC: citric acid soluble collagen.

Solubility of the AASC, LASC, and CASC samples was evaluated at different sodium chloride (NaCl) concentrations and various pH conditions. Higher solubility (more than 80% of relative solubility (RS)) was observed in all extracted collagens when treated with low NaCl concentration (0–10%). RS of >85% was recorded in the LASC sample (Figure 5A). This might have been due to the effect of the dialysis process in LASC that completely removed the remaining salt after being salted out during the precipitation process. As a result, no salt was detected in the lyophilized collagen. However, at high concentrations of NaCl (30 g/L to 60 g/L), the RS decreased sharply to less than 40% in all extracted collagens. Chen et al. [41] suggested that at high salt concentration, the hydrophobic–hydrophobic interactions in the polypeptide chain were escalated. The competition for water with salt ions was also increased, resulting in protein precipitation. These results were in accordance with collagens isolated from the skin and bone of Spanish mackerel (*S. niphonius*) [18], the skin of lizardfish (*S. tumbil*) [13], the skin and bone of golden pompano (*T. blochii*) [17], and the cartilage of Siberian sturgeon (*A. baerii*) [22].

In the context of pH, the solubility was increased in acidic acid conditions, especially at pH 1.0 and pH 5.0 (Figure 5B). The highest RS value (>90%) was noted in all extracted collagens treated at pH 3.0. In contrast, the solubility decreased at pH 7.0 and alkaline (pH 9.0) conditions. The increase in the hydrophobic–hydrophobic interactions among the collagen molecules might have resulted in the total net charge becoming zero, particularly at the isoelectric point which commonly occurs at slightly acidic and neutral conditions [54]. However, at pH 11.0, the RS were slightly increased to around 25–60%. This could be due to the effect of electrostatic repulsion between collagen molecules and hydration of charged residues at pH values above the isoelectric point (pI) [22]. Chuaychan et al. [22] stated that differences in the solubility of collagen treated with various pH were related to the difference in the molecular properties and conformations of collagen. Our findings were equivalent to those for the collagens extracted from the skin and bone of Spanish mackerel (S. niphonius) [18], skin of lizardfish (*S. tumbil*) [13], skin and bone of golden pompano (*T. blochii*) [17], and cartilage of Siberian sturgeon (*A. baerii*) [22].

## 3. Conclusions

Unicornfish (*N. reticulatus*) bone collagens extracted with the aid of various organic-acid solutions (i.e., acetic, lactic, and citric acids) were evaluated. The highest collagen yield (*p* < 0.05) was recorded for the citric-acid-soluble collagen (CASC) compared to that of acetic-acid-soluble collagen (AASC) and lactic-acid-soluble collagen (LASC). The triple-helical structures of type I extracted collagens were still maintained, indicating no denaturation of all samples during the acid-extraction process as confirmed by FTIR and XRD analysis. Although LASC had a lower collagen yield than CASC, other characteristics (i.e., thermostability, hydroxyproline content, color attributes, and solubility) were found to be preferable in the LASC sample. Thus, lactic-acid-soluble collagen (LASC) could be used as an alternative collagen for further research.

## 4. Materials and Method

### 4.1. Materials

Fifteen kilograms of fresh unicornfish (*N. reticulatus*) were obtained from a local supplier in Kota Kinabalu, Sabah, Malaysia. Samples were placed in an ice-cooled insulating box (ratio of fish to ice was 1:2 (*w*/*w*)) to maintain their freshness during transportation. Upon arrival, fish were subjected to species identification and weight (521.58 ± 32.27 g) and length (30.52 ± 4.28 cm) measurement. The prepared samples were separated automatically using a mechanical deboner machine (SFD-8 type, Taiwan). The fish bones were subsequently cut into small portions (1.0 × 1.0 cm^2^) with a stainless-steel knife (Brisscoes, Malaysia), and washed with running tap water. The washed samples were packed in a polyethylene bag and then stored in a freezer (−20 °C) for further analyses. Sodium dodecyl sulphate (SDS), acrylamide powder, Coomassie Blue R-250, *N,N,N′,N′*-tetramethyl ethylene diamine (TEMED), and Folin–Ciocalteu’s phenol reagent acetic acid (glacial) were purchased from Merck (Darmstadt, Germany). Precision Plus Protein Dual Color standards (markers) was purchased from Bio-Rad Laboratories (Hercules, CA, USA). Bovine serum albumin (BSA) and Lowry reagent were supplied from Sigma Chemical Co., (St. Louis, MO, USA). Citric acid and lactic acid solution were supplied from Systerm (Selangor Darul Ehsan, Malaysia) and Bendosen (Selangor, Malaysia), respectively. Other chemicals used in this research were of analytical grade.

### 4.2. Preparation of Acid-Soluble Collagen

**Figure 6:**
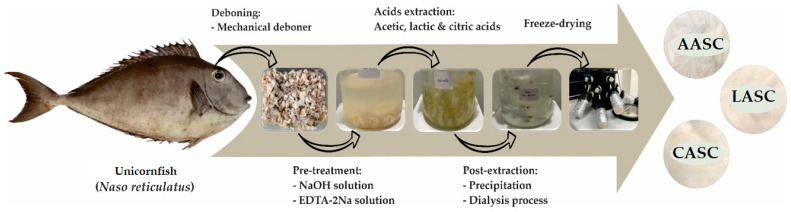
The procedure of acid-soluble unicornfish (*N. reticulatus*) bone collagen. AASC: acetic-acid-collagen; LASC: lactic-acid-soluble collagen; CASC: citric-acid-soluble collagen.

Extraction of unicornfish bone collagens with different acid solutions was carried out according to Jaziri et al. [13] with slight modification. All steps were strictly performed in a chiller (4 °C), and the extraction process is depicted in Figure 6. A total of 100 g fish bones was soaked in ten volumes of 0.1 M sodium hydroxide solution for 6 h (the solution was changed every 3 h) with continuous stirring. The pretreated samples were washed with distilled water to achieve neutral condition (pH 7.0). This step aimed to remove non-collagenous protein and pigmentation. Next, the neutralized samples were demineralized by dissolving in ten volumes of 0.5 M ethylenediaminetetraacetic acid disodium salt solution (pH 7.4) for 24 h with continuous stirring, and the solution was changed every 12 h. The demineralized samples were then washed with cold distilled water for 30 min and the distilled water was replaced every 10 min. After the pre-treatment step, the fish-bone samples were subjected to extraction by adding 15 volumes of 0.5 M organic-acid solutions (i.e., acetic acid, lactic acid, and citric acid) for 72 h. The extracted samples were then filtered through two layers of cheesecloth, and the filtrates were salted-out by dissolving in 2.5 M sodium chloride, followed by 0.05 M Tris (hydroxymethyl) aminomethane hydrochloride at a neutral pH (7.0). After that, the precipitates were centrifuged (Eppendorf Centrifuge 5810R, Hamburg, Germany) at 15,000× *g* for 15 min. The pellets were subsequently dissolved in acid solution (based on each acid used during extraction) at a ratio of 1:1 (*w*/*v*). The solutions were prepared for dialysis by transferring into a cellulose-membrane tubing (average flat width 43 mm, 1.7 in.) (Sigma-Aldrich, St. Louis, MO, USA) and then put in 15 volumes of 0.1 M acid solution for 24 h, followed by a distilled water for 48 h (the distilled water was changed every 24 h). The dialyzed samples were dried using a freeze-dryer machine (Labconco, Kansas City, MO, USA). After freeze drying, all dried samples were stored at a −20 °C. The dried collagens were labeled acetic-acid-soluble bone collagen (AASC), lactic-acid-soluble bone collagen (LASC), and citric-acid-soluble bone collagen (CASC).

### 4.3. Analyses

#### 4.3.1. Yield and Hydroxyproline (Hyp) Measurement

The yields of collagens (AASC, LASC, and CASC) were measured according to the formula developed by Jongjareonrak et al. [54], as noted below:

(1) $$\;\mathrm{Yield}\;\left(\%\right)=\frac{\mathrm{Weight}\;\mathrm{of}\;\mathrm{dried}\;\mathrm{collagen}}{\mathrm{Initial}\;\mathrm{weight}\;\mathrm{of}\;\mathrm{unicornfish}\;\mathrm{bone}}\times \;100$$

The Hyp content (mg/g) of extracted collagens was determined using an established procedure [55]. The lyophilized collagens were subjected to hydrolysis by adding a strong acid solution (6 M of HCl) at high temperature (110 °C) for 24 h. Afterwards, the hydrolysates were filtered through a Whatman filter paper No. 4 (Sigma-Aldrich, St. Louis, MO, USA). The filtrates were subsequently adjusted with 2.5 M NaOH solution to achieve pH of 6.5–7.0. Approximately 0.2 mL of hydrolysates was transferred into prepared test tubes and 0.4 mL isopropyl alcohol added. Subsequently, 0.2 mL of oxidant solution was added to the solutions and they were allowed to stand at room temperature for 5 min. After that, a total of 2.3 mL Ehrlich’s reagent solution was dropped in and mixed thoroughly. The mixtures were then heated in a water bath (Memmert, Schwabach, Germany) at 60 °C for 25 min. After heating, the treated samples were cooled for 10 min at room temperature. The cooled samples were further diluted with an isopropyl alcohol (up to 10 mL). Absorbance against distilled water was determined at a wavelength of 558 nm using a spectrophotometer (Agilent Cary 60, Santa Clara, CA, USA). Hyp standard solution (10 to 70 ppm) was also prepared in this current study.

#### 4.3.2. Color Attributes

Color attributes of AASC, LASC, and CASC samples were determined as described by Ismail et al. [56] using a colorimeter (ColorFlex CX2379, HunterLab, Reston, VA, USA). The attributes examined were L* (lightness), a* (redness: green to red), and b* (yellowness: blue to yellow). The whiteness index (WI) of the extracted collagens was calculated using the following formula [57].

(2) $$\mathrm{WI}=100\;-\;{\left[{\left(100\;-\;L^{*}\right)}^{2}+\left(a^{*2}\right)+\left(b^{*2}\right)\right]}^{0.5}$$

#### 4.3.3. Sodium Dodecyl Sulfate-Polyacrylamide Gel Electrophoresis (SDS-PAGE)

SDS-PAGE analysis of unicornfish bone collagens was conducted using a Mini-PROTEAN electrophoresis system (Bio-Rad Laboratories, Hercules, CA, USA). We used the established method from Laemmli [58] with minor amendments. Each dried collagen (around 2.5 mg) was dissolved in SDS solution (5%) and mixed thoroughly. The mixture was treated with high thermal condition (85 °C) using a water bath (Memmert, Schwabach, Germany) for 1 h. After heating, the mixtures were prepared by centrifuging at 8500× *g* for 5 min to remove undissolved matter. Around 15 µL of supernatants was transferred into a mini centrifuge tube and subsequently, 15 µL sample buffer in the presence and absence of 10% β-mercaptoethanol (β-ME) was added. After that, the mixtures were reheated at the same temperature for 5 min and then loaded into polyacrylamide gel composed of 4% stacking gel and 7.5% resolving gel. The acrylamide gel was electrophoresed with a constant voltage of 120 volts for 90 min. When electrophoresis ended, the gel was immersed in the staining solution containing 0.1% (*v*/*v*) Coomassie Blue R-250, 30% (*v*/*v*) methanol and 10% (*v*/*v*) acetic acid for approximately 30 min. Next, the stained acrylamide gel was destained with 10% (*v*/*v*) acetic acid and 30% (*v*/*v*) methanol solutions. The electrophoretic bands of AASC, LASC, and CASC were compared to the protein marker (Precision Plus Protein Dual Color Standards).

#### 4.3.4. Ultraviolet–Visible Absorption Spectra

Ultraviolet–visible (UV–vis) absorption spectra of all extracted collagens were determined using a UV–vis spectrophotometer (Agilent Cary 60, Santa Clara, CA, USA). A total of 5 mg of lyophilized collagens was dissolved with acetic acid solution (0.5 M), and well mixed. Next, the mixtures were centrifuged at 8500× *g* for 15 min to separate solubilized and insolubilized matters. The solubilized samples were then placed into a quartz cell (its optical path length was of 10 mm). The spectral wavelengths used in the present study were arranged from 400 nm to 200 nm with a baseline of acetic acid solution [14].

#### 4.3.5. Attenuated Total Reflectance–Fourier Transform Infrared Spectroscopy (ATR–FTIR)

ATR–-FTIR data of the AASC, LASC, and CASC were obtained from FTIR spectrometer (Agilent Cary 630, Santa Clara, CA, USA) as described from our previous study [15]. A total of 10 mg dried fish bone collagens was placed onto the crystal cell of FTIR spectrometer. Spectral data were prepared with a resolution of 2 cm^–1^ throughout a wavenumber range of 4000–1000 cm^–1^ for 32 scans against a background spectrum found from clean empty cells at ambient temperature. The obtained data were then analyzed using a software of Agilent Microlab.

#### 4.3.6. X-ray Diffraction (XRD) Data

Dried samples (AASC, LASC, and CASC) were scanned using a XRD instrument (Rigaku Smart Lab^®^, Tokyo, Japan), with copper Kα applied as an x-ray source. The tube voltage and current were set up at 40 kV and 50 mA, respectively. The scanning range in all acid-soluble collagens was prepared by adjusting from 10° to 40° (2θ) with a speed of 0.06° per second. The obtained data were recorded and analyzed by comparing to another previous research. XRD was carried out using the method of Chen et al. [21].

#### 4.3.7. Differential Scanning Calorimetry (DSC)

A DSC machine (Perkin-Elmer, Model DSC7, Norwalk, CA, USA) was used to obtain the thermostability value of the extracted collagens from the unicornfish bones. First, the prepared samples were hydrated by adding deionized water at a ratio of 1:40 (*w*/*v*) for 48 h in a chiller. The hydrates were then weighed from 5 mg to 10 mg into an aluminum pan (Perkin-Elmer, Norwalk, CA, USA), and tightly sealed. The DSC instrument was previously calibrated using an indium as a standard, and the sealed samples were subsequently scanned, ranging from 20 °C to 50 °C at a rate of 1 °C per minute. An empty pan was used as a reference. Thermostability in all samples was determined using the maximum transition temperature (*T_max_*), which was collected from the endothermic peak of thermogram, and the total denaturation enthalpy (*ΔH*) was recorded from the area of thermogram [59].

#### 4.3.8. Scanning Electron Microscopy (SEM)

Morphological evaluation of the AASC, LASC, and CASC samples was carried out using a scanning electron microscopy (Carl Zeiss, model MA 10, Germany). Prior to scanning, all acid-extracted collagens were sputter-coated with the gold for 5 min using a coater device (JEOL JFC-1200, Tokyo Rikakikai Co., Ltd., Tokyo, Japan). Next, all coated samples were imaged with a magnification (500×) [52].

#### 4.3.9. Solubility Study

Solubility of acid-soluble collagen was assessed at different sodium chloride (NaCl) concentrations and pH conditions. The procedure used was as described by Matmaroh et al. [59]. For solubility in NaCl, different NaCl concentrations (0–60 g/L) were used. Approximately 5 mL of solubilized collagens was prepared and transferred into 5 mL of different NaCl concentrations. The mixtures were then stirred continuously using a magnetic stirrer (ST0707V2, Selangor, Malaysia) for 30 min in a chiller. The mixtures were centrifuged (8500× *g*) for 10 min after being dissolved. For pH treatment, the dried collagens were added to 0.5 M of acetic acid solution. Afterwards, the dissolved samples were adjusted with different pH, from pH 1.0 to pH 11.0. The 2.5 N HCl and 2.5 N NaOH solutions were used for pH adjustment. The pH-adjusted samples were stirred for 2 h and subsequently centrifuged at 8500× *g* for 10 min. To determine percentage of relative solubility (% RS), all solubilized collagens (treated by different NaCl or pH) were subjected to protein measurement using the method of Lowry et al. [60], with bovine serum albumin (BSA) used as a standard. The RS (%) of all samples was measured using the following equation:

(3) $$\mathrm{Relative}\;\mathrm{solubility}\;\left(\%\right)=\frac{\mathrm{Current}\;\mathrm{concentration}\;\mathrm{of}\;\mathrm{protein}\;\mathrm{at}\;\mathrm{current}\;\mathrm{NaCl}\;\mathrm{or}\;\mathrm{pH}}{\mathrm{The}\;\mathrm{highest}\;\mathrm{concentration}\;\mathrm{of}\;\mathrm{protein}}\;\times \;100$$

### 4.4. Statistical Analysis

Experiment in this study was carried out in triplicate, and collected data are presented as means with standard deviation. One-way ANOVA was applied, and Duncan’s multiple range test was used to compare means with a significant effect signed in *p* < 0.05 under a SPSS Statistics version 28.0 (IBM Corp., Armonk, NY, USA).

## Figures and Tables

**Table 1:** Yield, Hyp composition, and color parameters of the acid soluble unicornfish (N. reticulatus) bone collagen.

Sample	Yield (%)	Hyp (mg/g)	Color Parameters			
			L*	a*	b*	WI
AASC	0.40 ± 0.15 ^c^	81.41 ± 0.11 ^a^	81.44 ± 5.25 ^a^	−0.19 ± 0.10 ^b^	0.79 ± 1.27 ^c^	81.37 ± 5.21 ^a^
LASC	1.08 ± 0.12 ^b^	81.32 ± 0.02 ^a^	82.55 ± 2.45 ^a^	0.40 ± 0.38 ^a^	6.51 ± 2.59 ^a^	81.28 ± 3.09 ^a^
CASC	1.36 ± 0.21 ^a^	80.17 ± 0.10 ^b^	79.35 ± 0.92 ^a^	0.04 ± 0.18 ^b^	3.26 ± 2.29 ^b^	78.97 ± 0.99 ^a^

AASC: acetic acid soluble unicornfish (*N. reticulatus*) bone collagen; LASC: lactic acid soluble unicornfish bone collagen; CASC: citric acid soluble unicornfish bone collagen. L* = lightness; a* = redness: green to red; b* = yellowness: blue to yellow; and WI = whiteness index. Different lowercase superscripts in the same column indicate significant difference (*p* < 0.05).

**Table 2:** The peak area and the description for the acid soluble unicornfish (*N. reticulatus*) bone collagen.

Peak Location			Peak Annotation
AASC	LASC	CASC	
3308.10 cm^−1^	3278.28 cm^−1^	3278.28 cm^−1^	Amide A, N-H stretching coupled with H bond
2920.44 cm^−1^	2924.17 cm^−1^	2924.17 cm^−1^	Amide B, CH_2_ asymmetric stretching
1638.21 cm^−1^	1638.21 cm^−1^	1617.71 cm^−1^	Amide I, C=O stretching/H bond coupled with COO-
1543.16 cm^−1^	1545.02 cm^−1^	1541.29 cm^−1^	Amide II, N-H bend coupled with C-N stretching
1237.51 cm^−1^	1237.51 cm^−1^	1235.64 cm^−1^	Amide III, N-H bend coupled with C-H stretching

AASC: acetic acid soluble unicornfish (*N. reticulatus*) bone collagen; LASC: lactic acid soluble unicornfish bone collagen; CASC: citric acid soluble unicornfish bone collagen.

**Table 3:** XRD and DSC analyses of the acid soluble unicornfish (*N. reticulatus*) bone collagen.

Sample	XRD Evaluation				DSC Data	
	1st Peak (Sharp Peak)		2nd Peak (Broad Peak)			
	2θ	d Value (nm)	2θ	d Value (nm)	*T_max_* (°C)	Δ*H* (mJ/g)
AASC	7.22	1.13	21.33	0.34	33.51	3.9
LASC	7.24	1.13	21.74	0.33	33.39	7.7
CASC	6.66	1.14	20.11	0.33	33.34	5.7

AASC: acetic acid soluble unicornfish (*N. reticulatus*) bone collagen; LASC: lactic acid soluble unicornfish bone collagen; CASC: citric acid soluble unicornfish bone collagen.

## Data Availability

The data presented in this study are available upon request from the corresponding author.
